# Identification and mapping of leaf, stem and stripe rust resistance quantitative trait loci and their interactions in durum wheat

**DOI:** 10.1007/s11032-012-9798-4

**Published:** 2012-10-20

**Authors:** A. Singh, M. P. Pandey, A. K. Singh, R. E. Knox, K. Ammar, J. M. Clarke, F. R. Clarke, R. P. Singh, C. J. Pozniak, R. M. DePauw, B. D. McCallum, R. D. Cuthbert, H. S. Randhawa, T. G. Fetch

**Affiliations:** 1 Semiarid Prairie Agricultural Research Centre, AAFC, Swift Current, SK Canada; 2 CIMMYT, 06600 Mexico, DF Mexico; 3 Department of Plant Sciences, University of Saskatchewan, Saskatoon, SK Canada; 4 Cereal Research Centre, AAFC, Winnipeg, MB Canada; 5 Lethbridge Research Centre, AAFC, Lethbridge, AB Canada

**Keywords:** Durum, Leaf, Stem, Stripe, Rust, QTL

## Abstract

Leaf rust (*Puccinia triticina* Eriks.), stripe rust (*Puccinia striiformis* f. *tritici* Eriks.) and stem rust (*Puccinia graminis* f. sp. *tritici*) cause major production losses in durum wheat (*Triticum turgidum* L. var. *durum*). The objective of this research was to identify and map leaf, stripe and stem rust resistance loci from the French cultivar Sachem and Canadian cultivar Strongfield. A doubled haploid population from Sachem/Strongfield and parents were phenotyped for seedling reaction to leaf rust races BBG/BN and BBG/BP and adult plant response was determined in three field rust nurseries near El Batan, Obregon and Toluca, Mexico. Stripe rust response was recorded in 2009 and 2011 nurseries near Toluca and near Njoro, Kenya in 2010. Response to stem rust was recorded in field nurseries near Njoro, Kenya, in 2010 and 2011. Sachem was resistant to leaf, stripe and stem rust. A major leaf rust quantitative trait locus (QTL) was identified on chromosome 7B at *Xgwm146* in Sachem. In the same region on 7B, a stripe rust QTL was identified in Strongfield. Leaf and stripe rust QTL around DArT marker *wPt3451* were identified on chromosome 1B. On chromosome 2B, a significant leaf rust QTL was detected conferred by Strongfield, and at the same QTL, a *Yr* gene derived from Sachem conferred resistance. Significant stem rust resistance QTL were detected on chromosome 4B. Consistent interactions among loci for resistance to each rust type across nurseries were detected, especially for leaf rust QTL on 7B. Sachem and Strongfield offer useful sources of rust resistance genes for durum rust breeding.

## Introduction

Durum wheat (*Triticum turgidum* L. var. *durum*) is an important food crop traded in the international market for use in pasta and couscous production. Durum wheat is cultivated on approximately 2 million hectares per year in the southern prairies of western Canada (Clarke et al. 2010), with a farm value of approximately $1 billion (Anonymous 2011).

Leaf or brown rust (*Puccinia triticina Eriks.*), stripe or yellow rust (*Puccinia striiformis* f. *tritici* Eriks.) and stem or black rust (*Puccinia graminis* f. sp. *tritici*) are some of the major constraints to durum wheat production. Leaf rust is globally distributed with diverse race structures that continuously evolve and form novel virulent races (Bolton et al. 2008; Kolmer 2005). Due to wide geographical distribution and frequent disease occurrences, leaf rust causes great annual losses (Huerta-Espino et al. 2011). Severe leaf rust incidence in a durum field can cause as much as a 70 % yield reduction (Herrera-Foessel et al. 2006). In addition to the direct yield losses, leaf rust causes quality downgrade, and additional costs are also incurred for disease control, e.g., application of chemical fungicides (Germán et al. 2007). Recent leaf rust epidemics on durum wheat in Mexico (2001–2009) caused significant economic impact, with a total loss estimated at about US$72 million (Huerta-Espino et al. 2011; Singh et al. 2004). Leaf rust epidemics have not been recently reported in Canadian durum due to past breeding efforts, which incorporated useful and effective resistance genes (Zhang and Knott 1990, 1993). However, emergence of new races requires continued efforts to deploy new leaf rust genes. The novel virulent leaf rust race BBG/BN and its variant BBG/BP overcame the resistance of widely adapted durum cultivars in North-western Mexico which had been effective and stable for more than 25 years (Huerta-Espino et al. 2009a, b; Singh et al. 2004). These virulent races pose a serious threat to durum cultivars of the USA and Canada because these races may spread through the North American rust corridor. Predominant Canadian durum cultivars are susceptible to BBG/BN and its variants including BBG/BP as determined by testing conducted in Mexico. This necessitates identification of sources of leaf rust resistance that can be bred into Canadian durum wheat.

Stripe rust is a destructive disease of wheat that can cause significant yield losses in severe epidemics due to reduction in kernel number and size (Ma and Singh 1996). The stripe rust pathogen is highly aggressive and variable on wheat, quickly evolving new races that overcome existing resistance (Chen 2005). Historically, stripe rust was widespread in the north-western USA and was typically associated with cool weather conditions for infection and development, but recently new races tolerant of higher temperatures were identified (Milus et al. 2009; Chen 2005), which poses a greater risk for spring wheat production in western Canada. Recent reports indicate that this pathogen has become a serious problem in all important Canadian wheat classes (Puchalski and Gaudet 2011; Randhawa et al. 2011a).

Under endemic conditions, stem rust is one of the most serious wheat diseases and has caused devastating damage to wheat crops worldwide over the past 100 years (Hodson 2011). Severe stem rust epidemics have occurred in North America and in Australia at continental levels leading to huge economic losses (Kolmer 2001; Park 2007). Genetic resistance has been the primary strategy to control stem rust in Canada. In the case of Canadian durum wheat, the stem rust 15-B resistant cultivar Stewart 63 was registered (Knott 1963) and additional sources were also transferred from emmer wheat (Knott 1962). Emergence of the new virulent race Ug99 in Uganda (Pretorius et al. 2000), and seven known races of Ug99 lineage that have spread to wheat growing countries in the eastern African highlands as well as Zimbabwe, South Africa, Sudan and Yemen, are posing a major threat to global wheat production because 90 % of the wheat varieties grown worldwide are susceptible to these new races (Singh et al. 2011). Resistance is available in some Canadian durum wheat cultivars (Fox et al. 2011), but the predominant cultivars are moderately susceptible.

Growing resistant cultivars is the most effective, sustainable and environment-friendly way of controlling rust diseases of wheat. Numerous studies have been conducted to identify sources of useful rust resistance in wheat, and over 68 leaf rust (McIntosh et al. 2011; Herrera-Foessel et al. 2012), 49 stripe rust (McIntosh et al. 2011) and 53 stem rust (McIntosh et al. 2011; Liu et al. 2011) resistance genes have been catalogued. The majority of these genes are identified and characterized in a hexaploid background. Rust patho-system and genetic determinants of resistance reaction differ between hexaploid and durum wheats, e.g., leaf rust (Martínez et al. 2007; Ordoñez and Kolmer 2007; Zhang and Knott 1990). Identification and mapping of rust resistance gene(s) in durum wheat is crucial for development of effective and durable host resistance in this crop. Quantitative trait loci (QTL) mapping studies can identify chromosomal regions with important traits and tightly linked markers that can then be used as an effective tool in marker-assisted selection (Collard et al. 2005). QTL mapping using high-throughput simple sequence repeat (SSR), single nucleotide polymorphism (SNP) or Diversity Arrays Technology (DArT) markers gives the opportunity for genome-wide mapping. QTL mapping has been utilized effectively to identify and map regions in the wheat genome that contain genes for leaf, stem and stripe rust. These include studies in multiple environments to determine the response of genotypes in different environments as well as to prevalent races.

Through preliminary studies, we identified that the durum wheat cultivar Sachem was resistant while Strongfield, the predominant cultivar grown on the Canadian prairies, was moderately susceptible to the BBG/BN leaf rust race and Ug99 stem rust races. Information on the genetics of resistance in Sachem and Strongfield is lacking for leaf, stem and stripe rust. The present experiment was carried out to determine the genomic regions or QTL governing leaf, stripe and stem rust resistance in the durum wheat genotypes Sachem and Strongfield, and to identify possible QTL interactions in the doubled haploid (DH) mapping population Sachem/Strongfield.

## Materials and methods

### Plant materials

A DH population comprising 71 lines from Sachem/Strongfield was developed at the Semiarid Prairie Agricultural Research Centre (SPARC) of Agriculture and Agri-Food Canada following the maize pollen method described by Knox et al. (2000). Sachem is a French genotype developed by Eurodur in France. Strongfield is a spring durum cultivar adapted to the semi-arid environment of the northern Great Plains (Clarke et al. 2005).

### Disease assessment

#### Leaf rust

The BBG/BN and BBG/BP leaf rust races and Ug99 stem rust and its variant races are not present in Canada, therefore disease testing was conducted in Mexico for leaf rust and in Kenya for Ug99. The parents and DH lines were evaluated for reaction to leaf rust in field nurseries operated by CIMMYT in Mexico near El Batan in 2009, Toluca in 2009 and 2011, and Obregon in 2010. Unreplicated rows of parents and DH lines were grown in El Batan, Obregon, and 2009 Toluca, while in 2011 near Toluca two replications were seeded. Field nursery rows were rated near anthesis. Each plot was visually rated for leaf rust severity following the modified Cobb Scale (Peterson et al. 1948). The parents and DH lines were tested for seedling response to races BBG/BN and BBG/BP in a greenhouse (GH) at CIMMYT, Mexico. For seedling response, the parents and DH lines were scored on the basis of scale consisting of infection types in the range 0, ;, 1, 2, X, 3, 4 (Roelfs 1984).

#### Stripe rust

The parents and DH lines were evaluated for reaction to stripe rust in field conditions at rust nurseries operated by CIMMYT in Mexico near Toluca in 2009 and 2011. Stripe rust reactions were also noted in 2011 from the Ug99 nursery grown near Njoro, Kenya. Unreplicated rows of parents and DH lines were assessed in 2009 (Toluca) and 2011 (Njoro), while in 2011 (Toluca) two replications per genotype were seeded and rated. In Toluca, each row was visually rated around anthesis for stripe rust severity as percentage of leaf covered with disease infection calculated as described for leaf rust. In Njoro, stripe rust infection type was rated based on a two-class infection response of resistant ‘R’ or susceptible ‘S’.

#### Stem rust Ug99 testing

Parents and DH lines were assessed for response to stem rust at Njoro, Kenya in 2010 and 2011. About 2 g seed per entry was planted in 2-m rows and spaced 30 cm apart. Urediniospores collected from lines containing the *Sr31* gene, and thus representative of “Ug99-type” races, were inoculated onto susceptible spreader rows. Response to stem rust was rated as stem rust severity (0–100 %) and infection response (R, R-MR, MR, M, MR-MS, MS, MS-S, S). Ratings were recorded around anthesis and repeated 2 weeks later.

### Molecular genotyping

The DNA was extracted from parents and DH lines for PCR using the Wheat and Barley DNA Extraction in 96-Well Plates protocol (http://maswheat.ucdavis.edu/PDF/DNA0003.pdf) with modifications. When the plants reached the 1–2 leaf stage, 3-cm leaf segments from primary leaves were harvested for genomic DNA isolation. A 10-μl PCR reaction consisting of DNA (final concentration of 20 ng/μl), Ultrapure Distilled H_2_O (Gibco), 10 %–10 × PCR Buffer without MgCl_2_ (Invitrogen), 10 mM dNTPs (Roche), MgCl_2_ 1.5 mM (Invitrogen), Taq 0.07 U/μ Taq (5 U of activity/μl) NEB, and 2 ng/μl forward and 2 ng/μl reverse primer was used for the DNA amplification process. PCR conditions were an initial denaturation at 94 °C for 3 min, followed by 44 cycles of 94 °C (1 min), 55 or 60 °C annealing (1 min) and 72 °C extension (1 min), with a final extension at 72 °C for 10 min. The annealing temperature for *Xgwm146* was 60 °C and for *wms344* was 55 °C. The amplification products were resolved by electrophoresis using a ABI3730xl DNA fragment analyser (Applied Biosystems), mixed 2 % Metaphor and 1 % agarose LE gels at 4 V cm^−1^ in TBE (0.045 M Tris, 0.045 M borate, and 0.001 M EDTA) buffer and stained with ethidium bromide (0.5 μg/ml). The size of bands was determined by comparison against a 50-bp DNA ladder. The DNA banding patterns were visualized with UV light and recorded by a Kodak Gel Logic 100 digital camera imaging system.

DArT genotyping was done by Triticarte Pvt. Ltd., Yarralumla, ACT, Australia (www.triticarte.com.au). DNA was extracted from parents and DH lines for DArT analysis according to the protocol published by Triticarte (http://www.triticarte.com.au/pdf/DArT_DNA_isolation.pdf). Briefly, a genomic representation of a mixture of the entire population was produced with *Pst*I-*Taq*I digestion, spotted on microarray slides, and the individual genotypes were screened for polymorphism based on fluorescence signals. A total of 257 polymorphic DArT loci were scored for the parental types.

### Statistical analysis

#### QTL analysis

Analysis of variance (ANOVA) was performed in SAS v.9.2 (SAS Institute 2010) to develop least square means for genotypes grown in 2011 near Toluca (experimental design Randomized Complete Block Design; genotype as fixed and replication as random variables). A genetic linkage map was constructed using the software JoinMap 4.0 using the regression mapping option and groupings were created using independence LOD (Van Ooijen 2006). Centimorgan (cM) values were calculated according to the Kosambi mapping function. Each linkage group was assigned to the corresponding durum wheat chromosome based on the known genomic positions of the DArT and SSR markers in the groups. QTL mapping was performed using MapQTL.6 (Van Ooijen 2009) to identify molecular markers significantly associated with leaf, stem and stripe rust resistance. The Kruskal–Wallis (KW) test (*P* < 0.001) and interval mapping (IM) were performed for each trait in each tested environment. Logarithm of odds (LOD) thresholds for significance were obtained by MapQTL’s permutation test option (1,000 permutations). Genome-wide threshold levels were used to declare significant QTL based on a 5 % significance level. Automatic co-factor detection based on backward elimination as well as manual co-factor selection was used to identify the co-factor markers for multiple QTL mapping (MQM).

#### QTL interaction analysis

QTL interactions were identified using the software Genotype Matrix Mapping (GMM) version 2.1 (Isobe et al. 2007, http://www.kajusa.or.jp/GMM). Briefly, in contrast to linkage mapping and association mapping techniques used to identify QTL, in GMM each marker analysed in a DH population is given a virtual matrix, known as the genotype matrix (GM). In the GM, intersecting lines and rows represent each of the total number of alleles for each marker locus which are used in estimating QTL interactions. The GMM method was used for selecting significant marker combinations consisting of two to three interacting markers using the ‘Automatic’ function for setting the Search Range. QTL interactions were studied for two to three loci for each trait, and significant locus combinations are reported on the basis of *F*-measure. Only the interactions with significantly lower disease means are reported. In the reporting of GMM results, ‘relevant samples’ refer to genotypes that have the interaction molecular variant combination significantly different from ‘others’, being all other molecular variant classes.

## Results

### Disease reaction

**Fig. 1:**
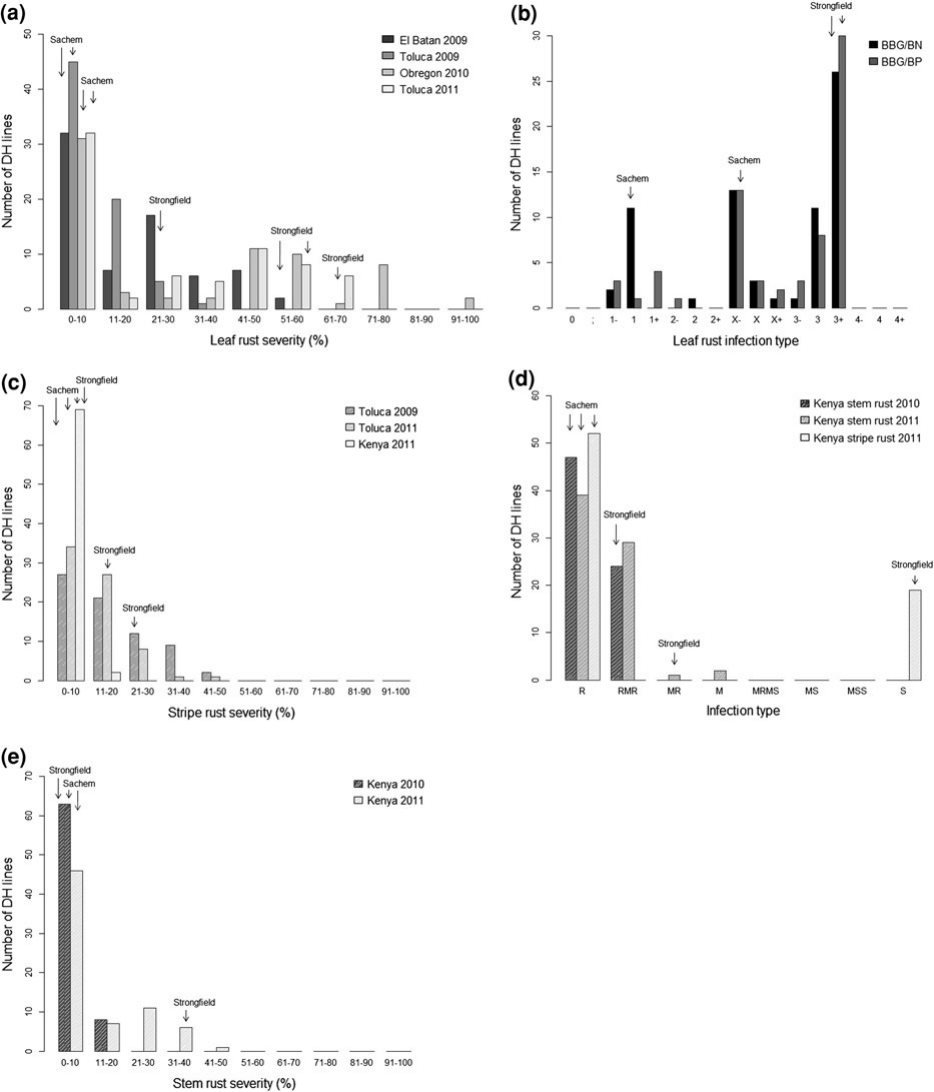
**a**–**e** Frequency distribution of disease ratings of the doubled haploid population derived from Sachem/Strongfield. **a**. Leaf rust severity (%) ratings were measured in field nurseries in 2009 near El Batan and Toluca, in 2010 near Obregon, and in 2011 near Toluca, Mexico; **b** screening for leaf rust races BBG/BN and BBG/BP in a greenhouse at CIMMYT, Mexico; **c** stripe rust severity (%) ratings were measured in field nurseries near Toluca, Mexico, in 2009 and 2011, and near Njoro, Kenya, in 2011; **d** stem and stripe rust infection type ratings were measured in specialized field nurseries created to screen for TTKS (Ug99) stem rust race reaction near Njoro, Kenya, in 2010 and 2011; **e** Stem rust severity (%) ratings were measured in specialized field nurseries created to screen for TTKS (Ug99) reaction near Njoro, Kenya, in 2010 and 2011

Leaf rust severity in field nurseries was 1 % at El Batan (2009), 5 % at Obregon (2010), 0 % at Toluca for Sachem (2009 and 2011) (Fig. 1). The leaf rust severity of Strongfield was 60 % at El Batan (2009), 70 % at Obregon (2010), 30 % at Toluca (2009) and 60 % at Toluca (2011). In the greenhouse tests (seedling response), the leaf rust infection type of Sachem was ‘1’ for BBG/BN and ‘X–’ for BBG/BP (Fig. 1b). Strongfield infection type to both of these races was ‘3+’. The DH lines varied in response from resistant to susceptible, with a larger proportion of DH lines showing low ratings in field nurseries (Fig. 1a). A larger proportion of DH lines showed high disease infection in GH testing (Fig. 1b). With a division of 2 + or less classified as resistant and greater than 2 + as susceptible, the DH lines segregated in a 1:3 ratio (Chi-squared goodness of fit *p* value = 0.37) to BBG/BN, while BBG/BP did not fit either a one- or two-gene ratio. The 10 DH lines with the lowest average leaf rust severity in the four nurseries were consistently low severity across the four leaf rust nurseries, while DH lines with high disease also had consistent expression in all nurseries. The same high disease expression lines were also susceptible in seedling reaction response.

Sachem had lower stripe rust severity than Strongfield in the tested environments. At Toluca, Sachem rated 10 % in 2009 and 7.5 % in 2011 (Fig. 1c). Strongfield rated 30 % in 2009 and 20 % in 2011. Stripe rust severity in 2011 near Njoro, Kenya was lower overall and Sachem rated 0 % and Strongfield rated 5 %. The response of DH lines was continuous and skewed toward lower disease severity. In the Njoro, Kenya nursery stripe rust infection response was different between Sachem (rated resistant) and Strongfield (rated susceptible), and DH lines also segregated as resistant and susceptible (Fig. 1c, d).

In the two stem rust Ug99 nurseries near Njoro, Kenya, Sachem had lower disease development than Strongfield. Sachem rated 0 % in 2010 and 7.5 % in 2011 (Fig. 1e). Strongfield rated 5 % in 2010 and 40 % in 2011. Infection response was resistant for Sachem in both years, while Strongfield rated R-MR in 2010 and MR in 2011. DH lines segregated between resistant and a moderate infection reaction (Fig. 1d, e).

Several DH lines had better disease response with the combination of leaf, stem and stripe rust resistance than either parent. In particular, DH line A0465&AG014 had less than 10 % leaf rust severity in all four field nurseries, ‘X–’ or better seedling reaction, 5 % or less stripe rust severity in the three stripe rust nurseries, and 10 % or less stem rust severity in the Njoro nurseries.

### QTL mapping

The DArT marker *wPt*-*0196* on chromosome 1A was significant for stem rust severity and infection response in 2011. Sachem possessed leaf and stripe rust resistance QTL on chromosome 1B at the marker *wPt*-3579. Significant stripe and leaf rust QTL in the region of DArT marker *wPt*-*3451,* unmapped to a linkage group in our population, were identified in Sachem. DArT marker *wPt*-*3632* on chromosome 2B was significant for leaf rust reaction in rust nurseries near El Batan and Obregon as well for seedling response to BBG/BN and BBG/BP, with the resistance deriving from Strongfield. DArT marker *wPt*-*3632* was also significant for stripe rust in 2011 (Toluca), with resistance derived from Sachem. On chromosome 4A, *wPt*-*4596* was significant for stem and stripe rust severity in 2011, with the Sachem molecular variant showing lower disease infection. The DArT marker *wPt*-*0872* on chromosome 4B was significant for Ug99 stem rust severity and infection type in 2010 and 2011. The same QTL was significant for stripe rust severity and infection type in 2011. DArT markers *wPt*-*6869* and *wPt*-*7219,* mapped at the same position but unlinked to other linkage groups in the Sachem/Strongfield population, were significant for leaf rust (Sachem-derived) in El Batan, Obregon, Toluca (2011), BBG/BN and BBG/BP, and stripe rust resistance (Strongfield-derived) in Toluca (2011). For leaf rust resistance, a major effect QTL was identified on 7BL with resistance derived from Sachem (Table 1). In the same region around *Xgwm146*, a stripe rust resistance QTL derived from Strongfield was identified in Toluca (2011).

**Table 1:** Summary of the significant markers identified through Kruskal–Wallis test in MapQTL software for leaf, stem and stripe rust from a doubled haploid population of Sachem/Strongfield screened for disease resistance for leaf rust (*Lr*) severity (%) in field nurseries (El Batan, Obregon, Toluca 2009 and 2011, Mexico) and greenhouse (seedling response infection type) at CIMMYT, Mexico (races BBG/BN and BBG/BP)

Locus	Chromosome number	K*	Significance	Mean sachem molecular variant	Mean strongfield molecular variant	Nurseries
*Xgwm146*	7B	49.73	*******	4.3	34.9	Lr-ElBatan2009
*Xgwm146*	7B	47.37	*******	4.2	57.3	Lr-Obregon2010
*Xgwm146*	7B	50.03	*******	3.5	47.1	Lr-Toluca2011
*Xgwm146*	7B	46.17	*******	7.4	13.6	BBG/BN
*Xgwm146*	7B	46.23	*******	8.5	13.8	BBG/BP
*Xgwm146*	7B	15.24	*******	18.0	9.3	Yr-Toluca2011
*wPt*-*0872*	4B	7.93	****	3.8	7.3	SrSeverity-Kenya2010
*wPt*-*0872*	4B	7.54	***	1.2	1.5	SrPustule-Kenya2010
*wPt*-*0872*	4B	12.87	******	6.4	18.6	SrSeverity-Kenya2011
*wPt*-*0872*	4B	9.79	****	1.4	1.7	SrPustule-Kenya2011
*wPt*-*0872*	4B	7.23	***	1.3	3.6	YrSeverity-Kenya2011
*wPt*-*0872*	4B	6.82	***	1.9	3.9	YrPustule-Kenya2011
*wPt*-*4596*	4A	7.90	****	6.3	16.0	SrSeverity-Kenya2011
*wPt*-*4596*	4A	8.07	****	1.2	3.4	YrSeverity-Kenya2011
*wPt*-*3632*	2B	10.13	****	26.3	11.7	Lr-ElBatan2009
*wPt*-*3632*	2B	8.93	****	41.0	18.5	Lr-Obregon2010
*wPt*-*3632*	2B	8.71	****	11.7	8.8	BBG/BN
*wPt*-*3632*	2B	7.54	***	12.1	9.9	BBG/BP
*wPt*-*3632*	2B	13.08	******	10.8	18.1	Yr-Toluca2011
*wPt*-*3579*	1B	7.20	***	10.6	16.6	Lr-Toluca2009
*wPt*-*3579*	1B	7.12	***	16.5	24.8	Yr-Toluca2009
*wPt*-*3579*	1B	10.96	*****	10.5	17.9	Yr-Toluca2011
*wPt*-*3451*	1B	10.03	****	9.3	16.1	Lr-Toluca2009
*wPt*-*3451*	1B	20.51	*******	12.4	26.1	Yr-Toluca2009
*wPt*-*3451*	1B	16.69	*******	8.7	17.2	Yr-Toluca2011
*wPt*-*0196*	1A	9.84	****	7.0	18.6	SrSeverity-Kenya2011
*wPt*-*0196*	1A	12.29	******	1.3	1.8	SrPustule-Kenya2011

Significance level (*P*-value): * = 0.05; ** = 0.01; *** = 0.001; **** = 0.0001 and so on

Stem rust (*Sr*) reaction (% severity and infection type) was recorded from Ug99 nursery near Njoro, Kenya, while stripe rust (*Yr*) ratings were collected from Toluca in 2009 and 2011 and Njoro, Kenya nursery in 2011

**Fig. 2:**
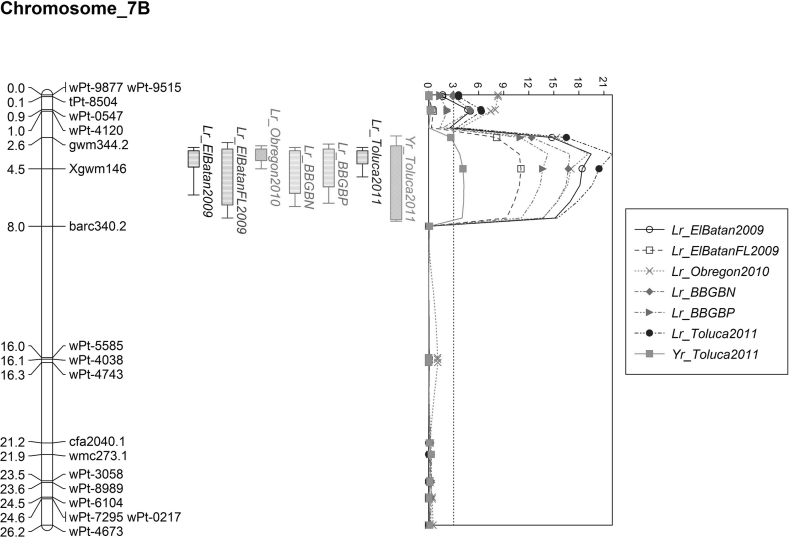
Identified QTL on chromosome 7B for stripe rust and leaf rust (major QTL and a minor QTL) using DArT and SSR markers using the Sachem/Strongfield DH population. Disease reactions were assessed near El Batan, Obregon and Toluca (2011), Mexico, field nurseries and indoor screening using leaf rust races BBG/BN and BBG/BP

The MQM analysis revealed a major QTL at marker locus *Xgwm146* on chromosome 7BL (Fig. 2). This QTL was significant for leaf rust seedling infection against BBG/BN and BBG/BP rust races and in leaf rust field evaluation nurseries near El Batan (2009), Toluca (2011) and Obregon (2010). In addition to the large effect QTL on 7BL, a smaller effect QTL with smaller peak was detected using the MQM analysis (Fig. 2). On 7BL, in addition to leaf rust, a significant stripe rust resistance QTL around *Xgwm146* was present where the Strongfield molecular variant conditioned resistance. The MQM analysis revealed a significant stripe rust QTL near *wPt3579* on chromosome 1B and stem rust QTL near *wPt*-*0872* on chromosome 4B with Sachem as the lower disease reaction molecular variant.

### QTL interaction analysis

**Fig. 3:**
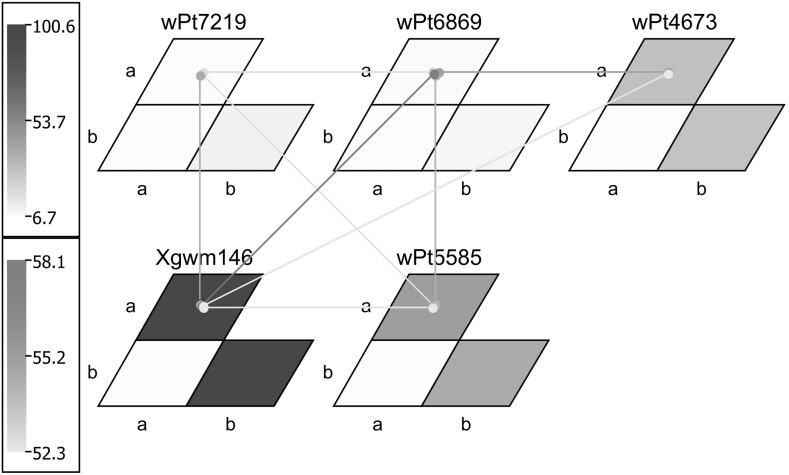
Graphical representation of QTL interactions for leaf rust infection in greenhouse for BBG/BP rust race in terms of matrices and connecting lines showing genotype matrices (GMs) and genotype matrix networks (GMNs). Significant locus/allele combinations of interacting loci are shown by GMs and GMN. Matrices and connecting lines indicate GMs and GMNs, respectively. Magnitude of the *F*-measure of combination and single locus/alleles effects, respectively, is shown by a color code

The QTL interaction analysis in GMM revealed that a significant two-loci interaction between DArT marker locus *wPt*-*6869*(aa) (unmapped in our study) and *Xgwm146*(aa) on chromosome 7B led to significantly lower leaf rust reaction in El Batan, Obregon, Toluca (2011), BBG/BN and BBG/BP (Table 2). ‘aa’ refers to Sachem alleles while ‘bb’ denotes Strongfield alleles at each marker locus. Other interactions that led to significantly lower disease infection included: for BBG/BP, reaction between DArT marker loci *wPt4673*(aa) and *Xgwm146*(aa) and loci *wPt5585*(aa) and *Xgwm146*(aa). The main effect QTL locus, *Xgwm146*(aa), contributed to the majority of the phenotypic variation in all leaf rust screenings. Figure 3 provides a visual depiction of BBG/BN QTL interactions identified in GMM. Significant two-loci interaction for stem rust (Kenya 2010) was observed between DArT loci *tPt5519*(aa) on 4B and *wPt9584*(bb) on 6A (Table 2). Stripe rust (Kenya 2011) significant two-loci interactions were identified between DArT loci *wPt3451*(aa) and *wPt2257*(aa) on chromosomes 1B and 1B/6B, respectively (Table 2). The QTL interactions for stem rust and stripe rust were governed by smaller effect QTL as demonstrated by the lower phenotypic variance explained (Table 2). Other interactions were determined that had significantly higher disease infection (data not presented).

**Table 2:** Significant positive QTL interactions for leaf, stem and stripe rust in disease nurseries near El Batan, Obregon, Toluca (2009 and 2011), Njoro, Kenya, and seedling resistance against BBG/BN and BBG/BP leaf rust races

Trait	Nursery	*F*-measure	Relevant samples		Others		Interacted locus (allele)	Phenotypic variance explained (%)
			# of Samples	Mean disease	# of Samples	Mean disease		
Leaf rust %severity	El Batan	233.2	32	2.9	38	35.3	*wPt6869*(aa)—*Xgwm146*(aa) Unlinked—Chrom. 7B	76.7
Leaf rust %severity	Obregon	173.2	32	2.5	38	57.4	*wPt6869*(aa)—*Xgwm146*(aa) Unlinked—Chrom. 7B	71.0
Leaf rust %severity	Toluca (2011)	240.1	32	2.2	38	47.2	*wPt6869*(aa)—*Xgwm146*(aa) Unlinked—Chrom. 7B	77.3
Leaf rust infection type	BBG/BN	95.1	32	6.9	38	13.3	*wPt6869*(aa)—*Xgwm146*(aa) Unlinked—Chrom. 7B	57.1
Leaf rust infection type	BBG/BP	58.1	32	7.9	38	13.3	*wPt6869*(aa)—*Xgwm146*(aa) Unlinked—Chrom. 7B	44.5
Stem rust severity	Njoro, Kenya (2010)	13.4	20	2.1	47	7.2	*tPt5519*(aa)—*wPt9584*(bb) Chrom. 4B—Chrom. 6A	14.5
Stripe rust severity	Njoro, Kenya (2011)	39.5	25	10.2	44	26.2	*wPt3451*(aa)—*wPt2257*(aa) Chrom. 1B—Chrom. 1B/6B	35.2

QTL interactions were identified using the software GMM on a double haploid population derived from Sachem/Strongfield. Relevant samples denotes the interaction class with significantly lower disease than the others (all other marker class combinations)

‘aa’ = Sachem alleles while ‘bb’ = Strongfield alleles at each marker locus

## Discussion

The Sachem/Strongfield population allowed segregation genetic analysis of leaf rust resistance. Field screening with a mixture of leaf rust races also enabled the study of genetics of rust resistance in Sachem and Strongfield. The continuous but skewed distribution of leaf rust reaction of DH lines at Toluca in 2009 indicated a poor environment for leaf rust resistance discrimination compared to the other three environments. The bimodal distribution in the other three environments indicated the influence of a major genetic factor on leaf rust disease response. The influence of a major gene is further supported by the distribution of DH lines between resistant infection types and susceptible infection types. The broad range of moderate severity to severe within the right-hand mode demonstrated the possible effects of additional minor genes. This is further supported the range in infection types within the resistant and susceptible classes and by QTL analysis with leaf rust genes identified on chromosomes 1B, 2B, 4B and 7B. Sachem contributed most of the resistance, with molecular variants for this parent having lower mean disease than Strongfield molecular variants for markers on 1B, 4B and 7B. In contrast, Strongfield possessed the leaf rust resistance allele on 2B.

Consistent with the qualitative segregation analysis, the major effect leaf rust QTL on 7BL was identified through KW and IM. We screened four SSR markers that had been reported to be present on this region of chromosome 7BL. Upon further dissection with SSR and DArT markers, MQM analysis suggested the possibility of a complex locus with two separate leaf rust resistance factors residing in the region of *Xgwm146,* with very tight linkage, i.e., a major leaf rust QTL, as well as a linked small effect QTL in the linkage group on the tip of chromosome 7B. *Xgwm146* has been reported to be linked to *Lr14a* (Herrera-Foessel et al. 2008). Our results indicate that Sachem possibly carries *Lr14a* and a tightly linked minor gene on 7B. Previously, two genes in close proximity have been suggested around *Lr14* (alleles *Lr14a*, *Lr14b*, and an uncharacterized gene) (Dyck and Samborski 1970). Schnurbusch et al. (2004) reported a minor leaf rust resistance locus on the distal end of the long arm of chromosome 7B, within a 5-cM region of an eight-marker cluster. Recently, Herrera-Foessel et al. (2012) reported a novel leaf rust resistance locus *(Lr68)* in close proximity to the previously mapped locus *Lr14a*, lending credence to our observations of two closely linked genes in Sachem on 7B. Our population size was sufficient to detect QTL, as indicated by the high degree of significance and multiple appearances over multiple environments for the stronger QTL, but a larger population will be needed to resolve the complex locus at *Xgwm146* and to determine whether Sachem carries both *Lr14a* and *Lr68*. Population sizes of the traditionally structured mapping populations such as recombinant inbred lines (RIL) are a function of the expected size of the QTL effect and the mode of inheritance (i.e., additive and dominance; additive in DH and RIL) (Lynch and Walsh 1997). As the effect of the QTL becomes smaller, a larger population is needed to increase the likelihood of QTL identification (Lynch and Walsh 1997). In our population, the marker positions agreed with published maps as well as identifying the major effect QTL. However, our population size is not large enough for more detailed resolution. Future work in a larger mapping population size will be required to better resolve the *Lr14a*/*Lr68* complex in Sachem.

The markers linked to the major QTL on 7BL in this study are common to those linked to the *Lr14a* gene reported in the Chilean durum cv. Llareta INIA (Herrera-Foessel et al. 2008) and the Italian durum cv. Creso (Maccaferri et al. 2008), and this resistance has been attributed to the *Lr14a* gene with the possible contribution of other minor genes (Maccaferri et al. 2008). *Lr14a* is not currently found in predominant Canadian durum cultivars, including Strongfield, therefore this gene is useful in Canada and can be deployed in combination with other *Lr* genes. However, given the widespread dependence on *Lr14a* in modern durum germplasm worldwide and since virulence for this gene has been reported in France to durum leaf rust races (Goyeau et al. 2010), the resistance from Sachem and other *Lr14a* sources should be used only in combination with other effective genes. With the development of markers to resistance genes such as *Lr14a*, marker-assisted selection can be used in pyramiding genes for increased durability.

On chromosome 1B, a leaf rust QTL was detected near *wPt*-*3451*. The DArT marker *wPt*-*3451* was previously reported to be significantly associated with a leaf rust seedling resistance gene on chromosome 1B (Nyori 2010) and other *Lr* genes have been reported in this region (McIntosh et al. 1995; Mago et al. 2002). Strongfield possessed a leaf rust resistance QTL on 2B (*wPt*-*3632*) in El Batan, Obregon, BBG/BN and BBG/BP evaluations, indicating that the small amount of resistance possessed by moderately susceptible Strongfield can be mapped. Several closely linked markers (*Xgwm410.1*, *Xgwm148* and *Xbarc183.1*) have been reported to be associated with *Lr* genes on the 2B linkage map: for example, *Lr16*, *Lr48*, *Lr13*, *Lr23* and *Lr50* (Nelson et al. 1997; Seyfarth et al. 2000; Bansal et al. 2008; Maccaferri et al. 2010).

A stripe rust resistance QTL was observed with Strongfield as the donor in the same region as *Lr14a*. It is not known if this is due to pleiotrophy or linkage. This region has been previously reported as a location of a *Yr* gene (Rosewarne et al. 2008; Suenaga et al. 2003). Li et al. (2009) also reported the presence of a single dominant gene *YrC591* on chromosome 7BL that was different from *Yr2* (McIntosh et al. 1998). In addition to stripe rust QTL on 7B, significant stripe rust resistance QTL were mapped near *wPt*-*3579*, on chromosome 1B, and *wPt*-*3451,* which did not map to any linkage group in our population but has been previously reported to be on 1B (Nyori 2010). The DArT marker *wPt*-*3451* is closely linked to *Xgwm413* on chromosome 1B, which is in a *Yr* gene-rich region including *Yr10*, *Yr15*, *Yr24*, *Yr26* and *YrH52* (Powell 2010; Ma et al. 2001; Peng et al. 2000). With the occurrence of new virulence in more aggressive races in 2011, the *Yr10* gene has been defeated in western Canada (Randhawa et al. 2011b). The detection of the 1BS QTL in Toluca but not in El Batan or Obregon, and the absence of the major 7BL QTL in Toluca, could be due to differences in predominant races. On chromosome 2B, the DArT marker *wPt*-*3632* was associated with resistance to leaf rust from Strongfield and was also significant for stripe rust resistance from Sachem, indicating the possibility of tightly linked *Lr*-*Yr* genes. The Strongfield association with leaf rust resistance and the Sachem association with stripe rust resistance indicates a repulsion linkage phase. Several stripe rust resistance genes (*Yr5*, *Yr7*, *Yr27*, *Yr31* and *Yr41*) have also been reported on chromosome 2B (Macer 1966; McDonald et al. 2004; McIntosh et al. 2006; Lou et al. 2008; Carter et al. 2009). Since we did not test SSR markers in the 2BS region, we cannot speculate on *Lr* and *Yr* genes that Strongfield or Sachem may possess. The sources of stripe rust resistance in Sachem are different from Strongfield, the current predominant durum cultivar in western Canada, and therefore offer additional options for stripe rust breeding.

QTL for stem rust severity on chromosome 4B and for infection type on 1A for Kenya 2011 were identified in this study. Stem rust gene *Sr37* has been reported on 4B (Loegering and Sears 1966; McIntosh and Gyarfas 1971) while more recently a gene controlling pseudo black chaff has been mapped in a similar region to the one we report (Bhavani et al. 2011). Further work is needed to confirm which genes are present in Sachem on 4B and if they are the same or different from *Sr37*. On chromosome 1A, *wPt0196* maps distant to the *Sr* QTL reported by Bhavani et al. (2011) and are likely distinct QTL (Trebbi et al. 2011; Akbari et al. 2006). These QTL in Sachem are useful in Canadian durum breeding programs because the predominant Canadian cultivars are susceptible to Ug99 and its variants (Fox et al. 2011).

Identification of significant positive (enhanced resistance) or negative (susceptible) QTL interactions are important in breeding for disease resistance. The significant interactions with lower disease response such as between DArT marker locus *wPt6869*(aa) and SSR marker locus *Xgwm146*(aa) on chromosome 7B provide the most value for improving disease resistance. However, the undesirable epistatic interactions leading to higher disease reaction are also important because they signify the combinations that can be avoided in a genome-wide selection approach. Our results demonstrate the importance of studying QTL interactions for rust resistance because even QTL that alone are not significant for trait expression can positively interact with other QTL to cause significantly better disease resistance. In breeding programs, such an approach would lead to more rapid gains. The genomic regions involved in QTL interactions were mapped using a new QTL mapping approach known as Genotype Matrix Mapping (Isobe et al. 2007) that has been used in other crops (Gautami et al. 2012; Ravi et al. 2011; Klimenko et al. 2010). An advantage of using this approach is that the interacting loci marker alleles can be shown in a graphical manner, making the complexity of QTL interaction more visual. Single locus QTL studied through GMM analysis gave similar results to MQM analysis. Our results indicated that the major portion of genetic variability for leaf rust involved interaction of a main-effect QTL (*Xgwm146*) with other significant loci. For stem and stripe rust, a significant proportion of the genetic variation for stem rust remained unanswered in our population, which could be due to sparse marker placement on the linkage map, to other undetected minor QTL, or modifier genes involved in this population contributing to a more complex inheritance. The combination of larger population size and a denser marker map would assist in obtaining more precision in QTL localization.

Since we used DArT markers to screen the DH population, they will need to be converted to markers amenable to marker-assisted selection in breeding laboratories (for example, PCR-based). Our results have identified genomic regions of interest but further genetic tests (including allelism tests) are necessary to confirm specific genes in Sachem and Strongfield. These rust resistance QTL in Sachem and Strongfield provide additional breeding options to durum breeding programs. The stem rust QTL on 1A and 4B are putatively novel and require further testing to confirm. Sachem possesses a unique combination of useful genes for leaf rust, stem rust and stripe rust, in addition Strongfield contributed useful leaf and stripe rust resistance QTL, making this DH population very useful for bringing together the genetic factors for rust resistance in the progenies. DH lines from this population are also good parental sources for simultaneously improving leaf, stem and stripe rust resistance.
